# Educating change agents: a qualitative descriptive study of graduates of a Master’s program in evidence-based practice

**DOI:** 10.1186/s12909-016-0597-1

**Published:** 2016-02-25

**Authors:** Grete Oline Hole, Sissel Johansson Brenna, Birgitte Graverholt, Donna Ciliska, Monica Wammen Nortvedt

**Affiliations:** Centre for Evidence-Based Practice, Bergen University College, Bergen, Norway; School of Nursing, McMaster University, Hamilton, ON Canada

**Keywords:** Evidence-based practice, Graduate education, Multi-professional education, Qualitative research, Learning organizations

## Abstract

**Background:**

Health care professionals are expected to build decisions upon evidence. This implies decisions based on the best available, current, valid and relevant evidence, informed by clinical expertise and patient values. A multi-professional master’s program in evidence-based practice was developed and offered. The aims of this study were to explore how students in this program viewed their ability to apply evidence-based practice and their perceptions of what constitute necessary conditions to implement evidence-based practice in health care organizations, one year after graduation.

**Methods:**

A qualitative descriptive design was chosen to examine the graduates’ experiences. All students in the first two cohorts of the program were invited to participate. Six focus-group interviews, with a total of 21 participants, and a telephone interview of one participant were conducted. The data was analyzed thematically, using the themes from the interview guide as the starting point.

**Results:**

The graduates reported that an overall necessary condition for evidence-based practice to occur is the existence of a “readiness for change” both at an individual level and at the organizational level. They described that they gained personal knowledge and skills to be “change-agents” with “self-efficacy, “analytic competence” and “tools” to implement evidence based practice in clinical care. An organizational culture of a “learning organization” was also required, where leaders have an “awareness of evidence- based practice”, and see the need for creating “evidence-based networks”.

**Conclusions:**

One year after graduation the participants saw themselves as “change agents” prepared to improve clinical care within a learning organization. The results of this study provides useful information for facilitating the implementation of EBP both from educational and health care organizational perspectives.

## Background

Health care professionals are expected to build decisions upon evidence. This requires that decisions are based on the best available, current, valid and relevant evidence from research, informed by clinical expertise and patient values [1]. Evidence-based practice (EBP) is seen as a core competence in several countries, but there are differences regarding the extent to which EBP is implemented [2].

One of the challenges in programs that teach EBP is to achieve the application of EBP skills in clinical care [3]. Integrating the teaching and learning of EBP as close to clinical practice as possible is recommended to assure sustainable learning for the participants [4, 5]. Ubbink et al. [6] published a systematic scoping review of 31 studies and found that 20 years after its introduction, EBP implementation in clinical care is still deficient. Young et al. [3] explored the effect of different teaching modalities to enhance the learners’ skills, knowledge and attitudes, practice and health outcomes, and in 2014 Ilic and Maloney [7] presented a review of 14 randomized trials regarding teaching EBP at different levels. Both reviews supported Horsley et al. [8] findings: the pathway from EBP education and training to using EBP competencies for improved clinical care is long and complex, and not well evaluated.

The effect of any postgraduate education on patient outcomes is debated [9]. Gijbels et al. [10] found low quality evidence in 61 studies of postgraduate education. Included studies measured mainly the students’ self-reported view of achieved competencies, but the competencies were not clearly defined. Cotterill-Walkers [11] carried out a comprehensive review to explore if Master’s education in nursing improved patient care. Participants reported constraints on their ability to practice new knowledge and skills. Another systematic review of the outcome and impact of a Master’s degree on health care revealed that the impact is seldom defined and mainly measured by self-reported surveys and qualitative studies [12]. There is a need for more in-depth studies of how participants in Master’s programs experience the impact on their professional practice, to ensure that the curriculum will meet the requirements from the clinical field.

The aims of this study were to explore how two cohorts of graduates of a multi-professional Master’s program in EBP a) perceived their ability to apply EBP one year after graduation and b) what they saw as necessary conditions to implement EBP at their workplace.

## Methods

To examine the graduates experiences a qualitative descriptive design was chosen [13, 14]. Within the pragmatic framework [15] focus group interviews were conducted to capture participants’ collective discussion and reflection, following Krueger and Casey’s guidance [16]. This included planning the study, development and refinement of the interview-guide (“the question route” ([16], p 41), moderating each interview, with use of appropriate “pauses and probes” ([16], p 99), using an iterative process of analyses, and interpreting data and presenting findings.

### Setting

Master’s programs in Norway are equivalent to two years full-time study, credited with 60 European Credit Transfer and Accumulation System (ECTS) credits each year [17]. A multi-professional Master’s program in evidence-based practice (MA-EBP) has been offered at Bergen University College, Norway since 2008. An overall aim of the program is to educate consumers of research who are able to initiate and carry out improvements in health care.

**Fig. 1:**
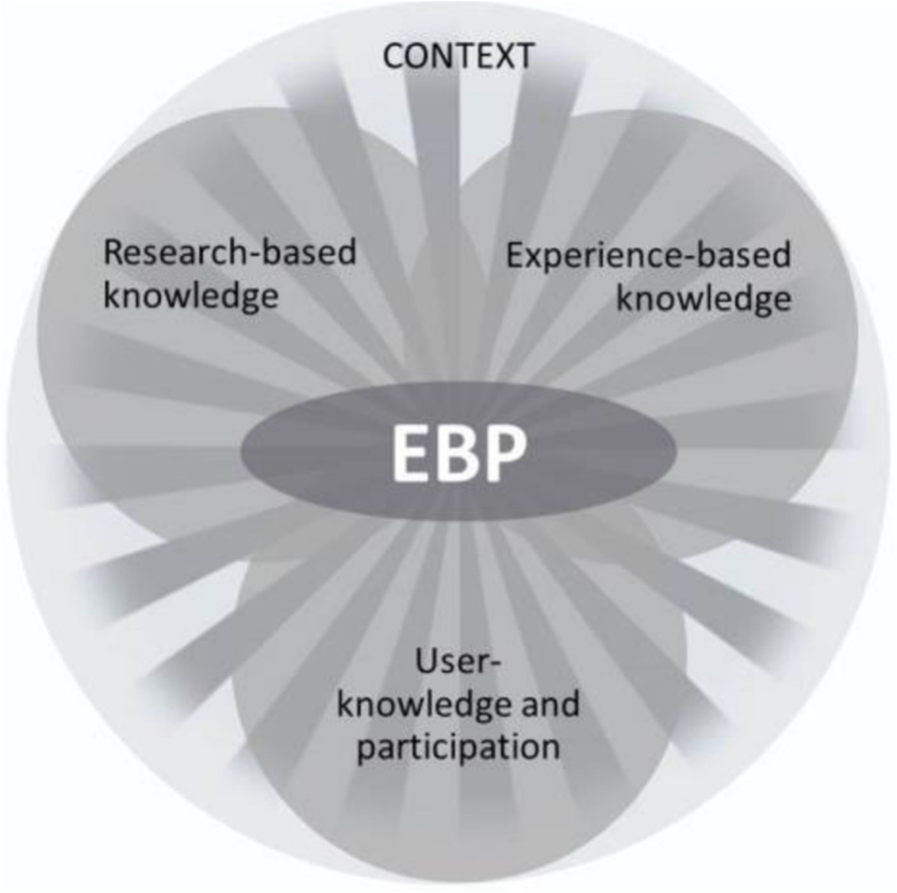
Model of evidence-based practice (EBP) [51]

The program follows a framework for EBP with a stepwise approach [1]. This includes utilizing evidence, knowledge from research, experience-based knowledge and the users’ knowledge and participation. These three sources of knowledge are used to inform clinical practice within a specific context, as presented in the model from the Norwegian Knowledge Centre for Health Services (Fig. 1).

The MA-EBP is a part-time study program. The first cohort received their 120 ECTS credits over three years. The second cohort, starting in 2009, gained the same number of credits over a four year program. The students were employed in different health care settings in Norway. They came to Bergen for week-long sessions three times each semester.

Students used their own work experiences to identify uncertainty in practice. These uncertainties served as starting points for every task and assignments throughout the program. For their thesis, most students either conducted a systematic review or evaluated current practice as a clinical audit.

The program itself is built upon evidence of how to teach EBP [6, 18] and is rooted in the concepts of adult, self-directed learning within a constructivist learning environment [19]. Adult learning theory is based on the premise that individuals learn what is relevant for them, building upon existing knowledge [20, 21]. The program encourages knowledge-sharing, learning and change within communities of practice [22].

The teachers supported the learners in a self-directed approach, to apply their skills and knowledge when they return to their workplace. By reflecting on what needs to be changed and how, the students were challenged to learn different strategies of addressing a problem. This included identifying barriers for change and opportunities to overcome these, and how to implement the necessary changes. Table 1 gives an overview of the Master’s program.

**Table 1:** Overview Master’s program in EBP

Semester	Topic	ECTS
1	Introduction to EBP in health care	15
2	EBP-Implementation I (clinical practice)	15
3	Philosophy of science, methodologies and methods	15
4	EBP-Implementation II (organization)	15
5	Developing a research project and in-depth research methods	15
6–8	Master’s thesis	45

Total of 120 European Credit Transfer System (ECTS)

4 years with 50 % study progression

Schön’s reflection in action is an underlying concept of this teaching modality [23]. Skilled health professionals build upon earlier experiences, link this to newly accomplished competencies from the program, then use those new competencies when they return to clinical work. Assignments and exams were completed between class sessions. Social interaction and collaborative small group learning were promoted to foster an environment where students could discuss and debate. Study groups met regularly both in and outside class, with interaction representing approximately 40 % of the learning sessions. Introductory lectures presented baseline knowledge, but these were interactive. Clinical scenarios were embedded in most learning activities.

### Sample and data collection

This study was conducted with the first two graduating cohorts (30 people). The graduates were mainly health care professionals but health librarians and health journalists were also represented. All had considerable work experience, and many had earned postgraduate education (Table 2). While enrolled in the program, students worked part-time and their work experience was essential for fulfilling some of the learning tasks.

**Table 2:** Demographic characteristics’ of participants

Informants	N 30/n 22
Age (mean)	29–56 (40.6)
Profession	
Registered Nurse^a^	16
Physiotherapist^a^	2
Occupational Therapist^a^	2
Health librarian/-journalist^a^	2
Further education	
Generic^b^	9
Inter-professional	3

^a^180 ECTS bachelor prepared

^b^90–120 ECTS post-graduate education

Invitations were sent by e-mail one year after graduation. Out of 30 potential participants, 22 volunteered, 11 from each cohort. Six semi-structured focus groups discussions with 3–4 participants in each were conducted. One participant who could not attend the focus group was interviewed by telephone.

The discussions were audiotaped and memos were written during the focus groups to capture the context. GOH and SJB facilitated and co-facilitated the interviews, except for one group in the second cohort where GOH had been supervising these students. In this case SJB moderated the discussion with another co-moderator.

The focus groups/interview lasted between 60–120 min. All professions represented in the cohorts participated in the focus groups and all participants were female. Each participant had a unique identifier, by the cohort (2008 or 2009), the sequence of the interviews (1–4) and by the number of the participants (1–4).

### Data analysis

The more than 12 h of taped discussion were transcribed to 130 pages of text by a research assistant. Interim analysis guided the planning of the next focus groups.

A thematic analysis was done with each transcript with the four themes for the semi-structured interview guide as starting point [24]. This focused upon experiences as students, perceptions of their competencies in EBP, role performance in EBP and suggestions for improvements in the educational program (not included in this paper as the purpose was feedback to the program). The analysis involved carefully reading through the transcript several times, first to get an overview over the interview and then to group the statements into the initial themes. When the three first interviews were grouped, a further rereading and grouping took place. The statements were then condensed and tentative codes and subcodes were developed for each group session. Thereafter the coded statements from the first cohort were pulled together for each initial theme. Next, statements were further grouped and condensed to new codes, where one sought to identify patterns and clarify what seemed to be essential for the students. Matrices were used to get an overview and make comparisons [25]. Saturation was not a goal of the analysis, as all potential participants were invited there was no possibility of increasing the number of participants.

The analysis was undertaken by GOH, but at each central step in the process the preliminary findings was discussed with SJB. Each session involved dialogue over the codes and how tentative findings could be interpreted. Reflection notes were written before and after the meetings. Each step followed the criteria by Lincoln and Guba to ensure trustworthiness and authenticity [26]. Findings from the first cohort guided the interviews and analysis with the second cohort. For the second cohort, we sought to explore more in-depth how the students experienced the intended link between the model of EBP (Fig. 1), the study program and the underlying pedagogical framework. Did the program promote the defined learning outcomes? Did it give participants the necessary competencies to implement EBP in their clinical practice? Minor adjustment of the interview guide and the introductory question were made at this point. Students’ experiences were still discussed, but with a closer focus on their feedback regarding the program rather than their personal experiences. For each theme we explored their experiences as adult learners, tasks rooted in cognitive learning theories linked towards lifelong learning and their thoughts about using EBP in the future. The coding first followed the same procedure as for cohort 1. Thereafter the findings from all seven data collection sessions were pulled together and further in-depth analysis was conducted with a closer focus upon the participants learning outcomes. Other authors (BG, DC and MWN) participated in analysis, discussion of the presentation of the findings and refinement of the model.

### Ethical considerations

The study was given ethical approval by the Norwegian Data Protection Official in accordance with the Personal Data Act and the participants gave their written informed consent. Efforts were taken to ensure that the participants were not recognizable in the presentation of findings.

## Results

**Fig. 2:**
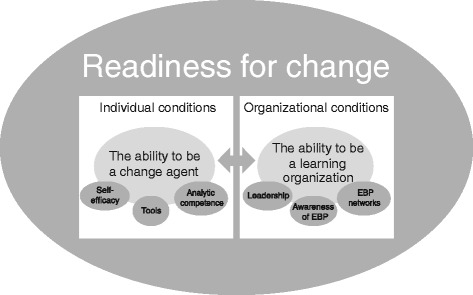
Model of “Readiness for change”

The aims of this study were to explore how the graduates perceived their ability to apply the principles of EBP and what they saw as necessary conditions to be able to follow the EB process in a clinical setting. Even though the study initially had focus upon the graduates’ individual capabilities, during the analysis it became clear that organisational factors were crucial for former students’ experiences during the MA-program and the first year after graduation. Individual factors were identified as “self-efficacy”, “tools” and “analytic competence” which together constituted an “ability to be change agents”. Factors related to the possibility of applying EB within their own organization were “leadership”, an “awareness of EBP” and “EBP networks”, which together provided the “ability to be a learning organization”. Together, this constituted the overall condition for EBP, and is the necessity of “readiness for change”, as illustrated in Fig. 2. While the participants appreciated the acquired knowledge and skills, organizational factors were crucial to their ability to utilize these competencies. The analysis revealed that these individual and organizational factors could not be seen as separate situations, but are mutually interdependent.

### The ability to be change agents

Participants viewed themselves as “change agents”. They stated that they had developed a strong aspiration for improving clinical care, including a firm desire for motivating and supporting others towards ‘best practice’ at all levels. *We must guide clinical practice towards the right way* several participants expressed. The right way meant judging every situation carefully, not slavishly follow guidelines or procedures. Three subthemes emerged within “ability to be a change agent”: “self-efficacy”, “analytic competence” and “useful tools”. These will be presented in turn.

### Self-efficacy

“Self-efficacy” was reflected by a raised consciousness of the informants’ professional standing, a firm belief in their abilities to promote best practice and an increased awareness of the importance of a reciprocal EBP-network.

Participants revealed a raised consciousness of their professional standing as a result of the constant reflection on their own practice and on the rationale behind clinical decisions, which the informants said was introduced by the program. These lead to improved professional confidence, independence and pride, and a increased awareness of professional conduct and diligent care. Statements as *I have increased pride in my work* and *I got a new view of my work, it is possible to improve clinical care* (08,2,2) indicates this. Participants described that the new knowledge was utilized in clinical reasoning with other professionals, which was appreciated by colleagues. *My mission is to ‘sow questions’ among my colleagues, - let them start to reflect* (08,1,2). They found that leaders and colleagues recognized their new skills, which gave them a new legitimacy.

They also expressed a firm belief in their own abilities to improve health services, and were confident that their effort could improve clinical care. By stating *I know I can make a difference* (08,2,1) participants demonstrated how they wanted to support their colleagues to work towards best practice.

### Analytic competence

“Analytic competence” referred to the ability to analyze a situation, describe it and argue for change. This “analytic competence” consists of both “organizational knowledge” and “awareness of the importance of relevant evidence for clinical decisions”.

The participants gave an impression of having gained theoretical and empirical understanding of the diversity and complexity of health care organizations, by statements such as *Health care systems are complex, with different cultures, but we learned how to overcome the difficulties* (08,1,1). This understanding made them able to identify key persons in their organizations who could be considered important opinion-leaders and stakeholders. *I became aware of how important it is to anchor suggestion of change with the leaders* (08,2,1).

A crucial part of “organizational knowledge” is the awareness of decision-making processes in health care organizations and the realization of their own abilities to influence decisions. Participants described that they knew and could use implementation strategies, theories of change, and how to identify and overcome barriers. In addition, they had confidence in their ability to position themselves at their own workplace to promote change. We also found participants describing improved organizational skills, the ability to work systematically and analytically, as well as improved communication and dissemination skills. During the program, students enhanced their appreciation of the importance of evidence-informed decisions. In line with the concept of EBP (Fig. 1), the participants reported that they used evidence from all sources of knowledge when discussing clinical issues. They also gained a scientific vocabulary which strengthened their ability to convince others and raised their credibility among colleagues. This was reflected in statements as *I will not just do as someone tells me, I want to know the reason for doing that* (08,2,2) and *I can argue for my opinion when I discuss with doctors, and my leader* (08,3,2).

### Useful tools for working with EBP

Participants expressed that the hands-on way of learning during the educational program gave them the competencies for promoting EBP. During the focus groups the graduates frequently stated *I have gained the necessary tools.* These were tools linked to the steps of EBP: the ability to reflect upon current practice, find relevant evidence from research, appraise the evidence and implement what they found.

Evaluating practice often exposed unwarranted variation and they were now able to argue for keeping or changing practice based on evidence from research and experience-based knowledge. They also found the language and vocabulary employed during discussions within the multi-professional group fostered a common language across different professional cultures. This made them able to argue with evidence both within their profession and across professions. *When you check what evidence there is, the discussions are better* (08,1,1).

### The ability to be a learning organization

Participants saw “the ability to be a learning organization” as a necessary condition for the implementation of EBP. Working with EBP is time-consuming, and it is important to decide who should do what, at what level within the organization and to allocate sufficient resources to achieve the use of evidence in changing practice. The participants described the necessity of a strategy for implementing EBP anchored in the organization. Several commented that all health care workers in Norway must know about EBP, but each individual need not follow the steps of EBP in their daily work. The implementation of EBP must have a systematic perspective, with dedicated persons promoting EBP at all levels within the organization, thereby building a learning organization. Several participants described their workplace as a learning organization, which allowed them to utilize their skills and knowledge gained during the program. Of those who did not have such support, some volunteered to engage in work with EBP, while others found new jobs where their new skills were more appreciated.

The subthemes identified under the organizations’ ability to be a learning organization were: “leadership”, “awareness of EBP” and “EBP networks”. Each will be presented below.

### Leadership

The data showed the importance of support from leaders. *My leader was very positive, and tasks I did according to the assignments are now implemented at my workplace* (08,2,3). This was followed by disappointment if the participants did not have such support. *Not one of my leaders was interested in my Master’s study. Never!* (08,2,2). They expressed the necessity for an organizational strategy for implementing EBP: *It is important that the leaders support the facilitation of EBP* (08,2,3). Such a strategy should be overarching for the health care organization and then be operationalized for each local workplace. Some informants perceived this where they worked. *Where I work all the leaders have committed to EBP* (08,1,3). But working in an EB way is time-consuming, and must be prioritized. Not all leaders recognized this. *It is difficult to work on EBP without added time and resources* (09,1,3).

### Awareness of EBP

Even though EBP is prized within Norwegian health care and is stated as an essential goal in several governmental papers [2], the discussion revealed that different levels of knowledge of EBP exist. Many statements indicated a lack of understanding of the concept. *My leader was very positive, but she did not know how to use what I learned* (08,2,1). Several of the participants used the phrase *There is a need for spreading EBP within clinical practice in Norway*.

The participants agreed that they used their new skills and competencies from the graduate program in their daily work, but they described the organizational learning attitude and motivation as central to their opportunities to do so. Some returned to their former workplace and were satisfied with their ability to use EBP, while others sought opportunities to work with EBP within their health trust. *I volunteered to write guidelines, must show them what I can do* (09,1,1). Quite a few of the graduates were engaged in new jobs, where their competencies are wanted and appreciated. For example, one participants stated *I now have a job where I can use what I learned every day. It is like doing my thesis once more* (09,3,2).

### EBP networks

Several participants expressed a need for EBP networks where they could share and support each other towards implementing EBP. During the program, peer students were important to them, and they wanted to keep contact after graduation. Statements like *The discussions in our group were essential for my learning* (08,3,1) and *There are so many resources among us, there is always somebody who knows more than me about an issue* (09,1,1) reflect this.

Returning to work they wanted to promote EBP. Their closest leader was central to success with this, but the participants also identified a need to assist their colleagues to implement EBP. *People ask me: do you know anything about this and that…? And it is satisfying to be able to find literature which answers the questions* (08,1,4). They raised the importance of being a part of a reciprocal EBP network, as a community with common goals. *As a student you could always ask someone for help. We still need networks after graduation, as we have a responsibility for implementing EPB* (08,1,1). This view was not only related to their workplace, but expressed as a need for a national EBP network.

## Discussion

The study sought to explore a) to what extent a new Master’s programme in EBP gave the students the ability to promote EBP and b) what the graduates saw as necessary conditions to implement EBP at their own workplace one year after graduation. The analysis revealed the importance of a “readiness for change” regarding implementation of EBP, as shown in Fig. 2. Participants experienced that the Master’s program in EBP gave them the ability to be a “change agent”. However, the importance of the organizations’ “ability to be a learning organization” was emphasized. Participants expressed that requirements for practicing their enhanced EBP skills were that the organization have “leadership which supports EBP”, an “awareness of the importance of EBP” within the organization and leaders who “support EBP networks”.

Compared to results of other studies within health [10, 12] it seems that participants in our study acquired more hands-on skills in promoting EBP and used their new skills to support a learning organization. While Cotteril-Walker’s review [11] clearly showed the constraints the students felt using new knowledge and skills, our informants confidently expressed that they could use what they learned during the Master’s program in their daily work. This might be due to how the Master’s program is constructed, leading students through the steps of EBP [1] within the framework of different sources of knowledge for decision- making (Fig. 1), and requiring them to use content related to organizational change and barriers for implementation. When planning the program, emphasis was put upon giving the students hands-on experience with implementation processes [27–29] during the program. In addition, one of the learning outcomes was that the students should become familiar with implementation strategies.

Several program characteristics may have contributed to the empowerment of the participants. In 2006, Khan and Coomarasamy [4] demonstrated the importance of interactive and clinically integrated teaching of EBP, and several others have shown the need for using different teaching modalities to stimulate the learning process [3]. The program structure, with assignments anchored in clinical issues from students’ own workplace, provided fertile learning with relevant and applicable knowledge. The participants felt this more strongly one year after graduation.

There were no traditional exams testing knowledge. Home-based assignments were constructed stepwise following the process of EBP [1], making room for cumulative learning over weeks. Discussion and collaboration between students was encouraged in a constructivists learning environment. Being one of the first Master’s programs for health professionals at that university college site, students had waited for some time for the initiation of the program and were highly motivated. Several participants stated that the assignments made them reflect upon why and how this had become the common way of working at their workplace. Reflection and discussions encouraged them to use information from all sources of knowledge (Fig. 1). They became more in touch with their tacit knowledge and learned to express what they knew from former experiences in a way other professions could understand. This is in accordance with the skill acquisition and articulation of embedded knowledge in expertise [30] and what is known of the development of professional practice [31], and clearly counters the traditional critiques of EBP as a cookbook-like practice where one slavishly follows instructions and procedures [32]. It seems like the hands-on training linked to the steps of EBP and the constant use of evidence from different sources gave the former students a solid foundation for practicing their knowledge and skills after graduation.

The curriculum is carefully created to enhance the students’ abilities to find and utilize evidence from research in clinical practice. How participants described the role as change agent has also been labelled as “knowledge brokers” [33], as they were able to understand the clinical questions, find and critique relevant research and implement the findings when appropriate to their clinical unit. Knowledge brokers are one of many knowledge translation (KT) strategies used during the last decade [34]. A randomized trial of three levels of KT intervention intensity [35] revealed the importance of knowledge brokers to promote evidence into practice, particularly when the organizational culture for research use was low, which has been the case in many Norwegian health care institutions.

To retain staff with these skills, it was important that the health care services were learning organizations. Learning organizations have been described in the leadership literature over the last decades as important for innovation and change. A learning organization is structured to facilitate creation as well as sharing of knowledge among members or employees, and to foster individual skills as an important part of the organization’s collective competence [22]. In United Kingdom it was considered an important part of the new strategy for the National Health Service [36–38] as well as a necessary condition to address the ‘knowing-doing gap’ when promoting EBP [39]. A systematic review summarizing how to spread and sustain innovations in health services delivery emphasised the need for a knowledge-based approach to innovations in organizations, according to the goals and values of a learning organization [28]. In our study, leadership was considered an important factor for the implementation of EBP in learning organizations, as leaders were able to clearly prioritize and mobilize resources.

The necessity of leadership and resource allocation was also found in a case study by Peirson et al. [40], who studied a public health unit’s long-term strategic initiative to build organizational and staff capacity for evidence informed decision-making. The leaders at the highest levels were the ones who “stimulate and propel change” ([40], p 9). Leaders with vision and mission were essential in setting the course of change. It is important that both leaders and the structure of the organization make room for knowledge brokers to do their work, and allow time for participation in supportive networks. This is consistent with findings from Melnyk et al. [41, 42]; which emphasized the strong need for incorporating organizational change as well as leadership when planning the implementation of EBP.

Participants reported that their role as “change agents” became an important part of the organizational network supporting EBP, where new individual skills contributed to the enhanced competencies in the organization. This is in line with findings from other studies pointing at the importance of the culture of the organization [22, 28, 35, 37, 39], and is further supported by Michie et al. [27] with the identification of different domains within psychological theories. The domains of “environmental context and resources” and “social influences” ([27], p 30) demonstrate the importance of of the culture of the organization when implementing EBP.

The growing focus on quality in health services link EBP as a necessary premise and tool for ensuring best practices. As early as in 2001 it was one of the ten recommendations in Institute of Medicine’ seminal report “Crossing the Quality Chasm: A New Health System for the 21^st^ Century” [43]. The qualities of learning organizations, lifelong learning, leadership and structural components to support EBP are seen as crucial components to raise quality of care in the Magnet Hospital Recognition Program [44–47]. Our study results show that participants discovered the same factors to be important in their ability to apply EBP. The close interplay between individual and organizational factors which constitutes a “Readiness for change” are in line with facilitators presented elsewhere [48, 49]. As demonstrated by Melnyk et al. [41, 42], as well as findings from Williamson et al. [50], these factors are very important when planning educational interventions to promote EBP and supporting clinical leaders.

### Strength, limitations and research implications

The present study is a small study with only two cohorts and 22 participants. However, there was a high participation rate from the total of 30 potential participants. The focus groups triggered reflections of learning, and perceptions of their ability to EBP in the work situation. We sought to avoid the domination of any few participants with the use of a co-moderator to ensure that all participants could take part in the discussion. The interview guide worked well. After each interview the interim analysis started, and tentative findings guided the next interviews. A follow-up study with in-depth interviews with the graduates would be useful to explore how they overcame barriers to implementing EBP, how they contributed to building a learning organization; and what difference the implementation of EBP made on clinical outcomes.

## Conclusion

Our findings demonstrated that one year after graduation, the participants reported that the Master’s program of EBP had given them applicable knowledge they found useful in their daily work. Together with the acquired organizational knowledge and analytic competencies, they saw themselves as “change agents”, prepared to improve clinical care within a learning organization.

If dedicated professionals are to succeed in improving patients’ outcomes’, both organizational and individual factors must be addressed. The findings from our study indicate that the Master’s program led to the intended learning outcomes and gave the graduates the necessary competencies to implement EBP in their own workplace if the factors important for creating a “readiness for change” are present. This knowledge may prove valuable for facilitating EBP in health care, and can be guidance to the developers of EBP curriculum.

## Abbreviations

EB: evidence-based
EBP: evidence-based practice
ECTS: European Credit Transfer and Accumulation System
KT: knowledge translation
MA-EBP: Master’s program in evidence-based practice
